# Effect of crocin on antioxidant gene expression, fibrinolytic parameters, redox status and blood biochemistry in nicotinamide-streptozotocin-induced diabetic rats

**DOI:** 10.1186/s40709-020-00114-5

**Published:** 2020-03-02

**Authors:** Ioannis Margaritis, Katerina Angelopoulou, Sophia Lavrentiadou, Ilias C. Mavrovouniotis, Maria Tsantarliotou, Ioannis Taitzoglou, Alexandros Theodoridis, Aristidis Veskoukis, Efthalia Kerasioti, Dimitrios Kouretas, Ioannis Zervos

**Affiliations:** 1 Laboratory of Physiology, School of Veterinary Medicine, Faculty of Health Sciences, Aristotle University of Thessaloniki, Thessaloniki, Greece; 2 Laboratory of Biochemistry & Toxicology, School of Veterinary Medicine, Faculty of Health Sciences, Aristotle University of Thessaloniki, Thessaloniki, Greece; 3 Private Diagnostic Laboratory, Diavata, Thessaloniki, Greece; 4 Laboratory of Animal Production Economics, School of Veterinary Medicine, Faculty of Health Sciences, Aristotle University of Thessaloniki, Thessaloniki, Greece; 5 Department of Biochemistry and Biotechnology, University of Thessaly, Viopolis, Mezourlo, 41500 Larissa, Greece

**Keywords:** Saffron, Diabetes, PAI-1, Antioxidants, Crocin, Rats

## Abstract

**Background:**

Diabetes is regarded as an epidemiological threat for the twenty-first century. Phytochemicals with known pharmaceutical properties have gained interest in the field of alleviating secondary complications of diseases. Such a substance is crocin, a basic constituent of saffron (*Crocus sativus*). The present study aimed at examining the beneficial effects of per os crocin administration on the antioxidant status, blood biochemical profile, hepatic gene expression and plasminogen activator inhibitor-1 activity (PAI-1) in the liver, kidney and plasma (an important marker of pre-diabetic status and major factor of thrombosis in diabetes) of healthy rats, as well as of rats with nicotinamide-streptozotocin-induced diabetes.

**Results:**

Diabetes disrupted the oxidation-antioxidation balance, while crocin improved the antioxidant state in the liver by significantly affecting SOD1 gene expression and/or by restoring SOD and total antioxidant capacity (TAC) levels. In the kidney, crocin improved hydrogen peroxide decomposing activity and TAC. In blood, hepatic transaminases ALT and AST decreased significantly, while there was a trend of decrease regarding blood urea nitrogen (BUN) levels. The expression of PAI-1 gene was affected in the liver by the dose of 50 mg kg^−1^.

**Conclusions:**

Crocin treatment contributed in restoring some parameters after diabetes induction, primarily by affecting significantly hepatic transaminases ALT and AST, SOD1 and PAI-1 gene expression and nephric H_2_O_2_ decomposing activity. In conclusion, crocin did contribute to the alleviation of some complications of diabetes.

## Background

Diabetes mellitus has been characterized as an “epidemiological threat” of the twenty-first century [1]. In 2017, it was estimated that there had been 425 million of diabetics worldwide, while 212 million of them were considered undiagnosed [2]. The relationship between diabetes and oxidative stress is well established and reactive oxygen species (ROS) are involved in its pathophysiology [3]. Furthermore, disruption of carbohydrate, protein and fat metabolism due to oxidative stress results in complications, such as micro- and macrovascular dysfunctions [4].

A growing research effort is being made regarding the usage of substances of herbal origin, such as resveratrol and quercetin [5], for the reduction of oxidative stress associated with the disease [6]. Another substance used is crocin (crocetin di-gentiobiose ester), one of the most significant active compounds of the plant *Crocus sativus* L. and of the respective elegant spice, saffron. It is a compound reported to possess potent antioxidant [7] and—possibly—hypoglycemic properties [8].

Plasminogen activator-inhibitor-1 (PAI-1), a single chain glycoprotein of 379–381 amino acids, is mainly produced in endothelial and smooth muscle cells (fibers), although other cells like platelets, mesangial cells, fibroblasts, hepatic cells, monocytes and adipocytes produce the inhibitor as well [9]. It is the main inhibitor of plasminogen activation and its increased concentration in plasma promotes clot formation, which is related to cardiovascular disorders, an element observed in obesity and type-2 diabetes [10]. Accumulating evidence correlates increased PAI-1 levels with insulin resistance and type 2 diabetes [9], although hyperglycemia *per se* can increase PAI-1 secretion as well [11]. Recently, in a lipopolysaccharide (LPS) animal model of thrombosis, it was shown that crocin can acutely suppress PAI-1 activity [12]. Since some of the observed effects did not correlate with alterations of the oxidative status, the authors suggested that this effect must be due to a different, probably direct mechanism that may involve currently unknown properties of the crocin molecule that go beyond its antioxidant capacity. Feidantsis et al. [13] also made a similar, yet not substantiated suggestion in a study on the effect of crocin on cardiac dysfunction in STZ-induced diabetic cardiomyopathy.

The objective of our study was to examine the potential ameliorating effects of crocin supplementation regarding the disruption of the oxidative status by the diabetic state. For this purpose, we carried out blood biochemical tests and determined a series of oxidative stress markers in the liver and the kidney. The liver plays a major role in metabolism as the main site for gluconeogenesis, while the antioxidant status of the organ after diabetes induction is of major importance. We also measured PAI-1 activity and gene expression in the liver, a principal source of the enzyme [9], along with the determination of antioxidant enzyme gene expression, and correlated the results with the respective parameters referring to the redox status of the organ. As for the kidney, apart from being a main organ for drug metabolism, diabetic nephropathy is a complication of diabetes of high research interest. Finally, we investigated the effect of crocin supplementation on the expression of certain hepatic genes that encode antioxidant proteins, namely SOD1, SOD2, and catalase, as well as on PAI-1 gene expression and PAI-1 activity in blood plasma and kidney.

## Results

### Animal weight

Animal weight was significantly decreased in the diabetic control group compared to the controls (*p* < 0.05), while crocin administration did not compensate for this effect (Table 1).

**Table 1:** Body weight and serum glucose concentration (mean ± SD)

	C	Cr20	Cr50	D	DCr20	DCr50
Weight (g)	310 ± 26.3^a^	277.8 ± 17.9	291.3 ± 7.6	233.8 ± 22.2^b^	226.3 ± 26.3	212.7 ± 38.4
Glucose (mg dl^−1^)	164.8 ± 15.2^a^	141.7 ± 12.8^c^	128.8 ± 11.9^c^	584.8 ± 119.6^b^	666.7 ± 104.7	565.2 ± 96.9

Different letters in the same row represent statistically significant differences (*p* < 0.05)

### Effect of crocin on biochemical parameters in blood serum

Glucose in serum decreased by the administration of crocin to non-diabetic animals. The effect, already significant at the dose of 20 mg kg^−1^ (*p* = 0.025), was even more clear at the higher crocin dose (*p* = 0.01). Furthermore, serum glucose was substantially increased by the diabetic state (*p* < 0.01) but crocin administration did not compensate for this increase (Table 1).

**Fig. 1:**
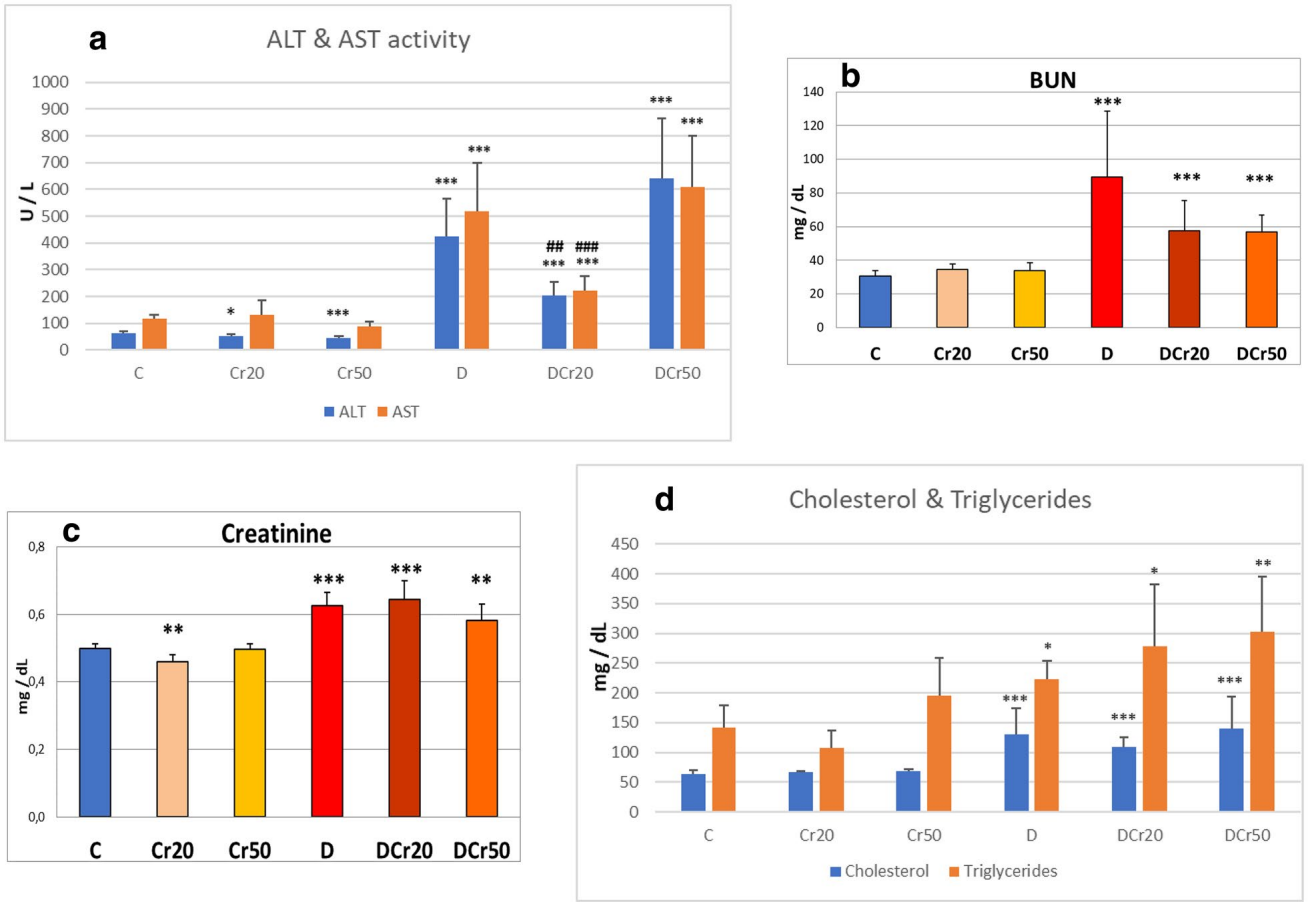
**a** ALT and AST activity in serum (mean ± SD). *(*p* < 0.05), ***(*p* < 0.005): statistically significant compared to the C group; ^##^(*p* < 0.01), ^###^(*p* < 0.005): statistically significant compared to the D group. **b** BUN concentration in serum (mean ± SD). ***Statistically significant compared to the C group (*p* < 0.005). **c** Creatinine concentration in serum (mean ± SD). **Statistically significant compared to the C group (*p* < 0.01), ***Statistically significant compared to the C group (*p* < 0.005). **d** Cholesterol and triglyceride concentration in serum (mean ± SD). *(*p* < 0.05), **(*p* < 0.01), ***(*p* < 0.005): statistically significant compared to the C group

The activity of alanine aminotransferase (ALT) was decreased by the administration of crocin to healthy animals, in a dose dependent manner (*p* = 0.024 for the 20 mg kg^−1^ dose of crocin, and *p* = 0.004 for 50 mg kg^−1^ of crocin). Additionally, ALT activity was markedly increased in the diabetic animals compared to the control group (*p* < 0.01) while crocin administration at the dose of 20 mg kg^−1^ induced a decrease of the enzyme activity compared to diabetic controls (*p* < 0.01). On the other hand, the 50 mg kg^−1^ dose of crocin did not have any similar effect (Fig. 1a).

Regarding aspartate aminotransferase (AST) activity in serum, the higher dose of crocin resulted in strong trend of decrease in non-diabetic animals, only marginally not significant (*p* = 0.065). Diabetes affected AST activity in serum. Specifically, there was a marked increase of the enzyme activity compared to the control group (*p* < 0.01), while the administration of crocin to diabetic animals at the dose of 20 mg kg^−1^ caused a statistically significant decrease (*p* < 0.01). On the other hand, the result was not similar for the DCr50 group (Fig. 1a).

With respect to blood urea nitrogen (BUN) concentration, it was increased in the D group compared to the controls (*p* < 0.01). Crocin administration to diabetic rats did not reverse this effect in a statistically significant way, yet it is intriguing that the *p* values were low for both doses tested (*p* = 0.107 and *p* = 0.128 for DCr20 and DCr50 groups, respectively) (Fig. 1b).

Creatinine concentration was reduced (*p* < 0.01) by the administration of the lower dose of crocin to healthy rats. With respect to diabetic animals, it was significantly increased in the D group compared to the controls (*p* < 0.01). On the other hand, the administration of crocin did not alleviate this effect (Fig. 1c).

As for cholesterol concentration, it was significantly increased in the D group compared to the control group (*p* < 0.01), but crocin intake did not counterbalance this effect (Fig. 1d).

Diabetes caused a statistically significant increase of triglyceride concentration compared to the control group (*p* = 0.01), and crocin intake did not compensate for this effect (Fig. 1d).

### Effect of crocin on redox biomarkers

#### Hydrogen peroxide decomposing activity

Hydrogen peroxide decomposing activity in the liver did not change significantly by the supplementation of crocin to healthy animals, neither did it change in the diabetic rats (Table 2).

**Table 2:** Redox biomarkers in the liver (mean ± SD)

	C	Cr20	Cr50	D	DCr20	DCr50
H_2_O_2_ decomposing activity (U mg^−1^ protein)	377.57 ± 67.58	391.39 ± 74.64	378.68 ± 47.64	357.27 ± 97.14	473.94 ± 143.44	353.5 ± 113.26
SOD (U mg^−1^ protein)	33.9 ± 3.55^a^	34.65 ± 10.88	69.56 ± 15.89^b^	50.8 ± 13.4	59.1 ± 17.26	34.28 ± 10.21
GSH (μmol mg^−1^ protein)	0.036 ± 0.006^a^	0.035 ± 0.007	0.072 ± 0.024^b^	0.042 ± 0.009	0.056 ± 0.023	0.044 ± 0.014
Protein carbonyls (nmol mg^−1^ protein)	1.44 ± 0.62	2.81 ± 1.4	3.12 ± 0.98	3.05 ± 1.31	3.3 ± 1.37	1.9 ± 1.1
TΑC (mmol DPPH mg^−1^ protein)	0.160 ± 0.02^ab^	0.13 ± 0.03	0.259 ± 0.08^c^	0.227 ± 0.07^bc^	0.215 ± 0.08	0.13 ± 0.04^a^

Different superscripts in the same row represent statistically significant differences (*p* < 0.05)

In the kidney, hydrogen peroxide decomposing activity demonstrated a different pattern. Specifically, it was significantly reduced in the Cr50 group (*p* = 0.004) and the diabetic group (*p* = 0.004). Interestingly, the administration of crocin to diabetic rats at the dose of 20 mg kg^−1^ ameliorated this effect (*p* < 0.05), but it still did not restore the activity to the level of the control group (Table 3).

**Table 3:** Redox biomarkers in the kidney (mean ± SD)

	C	Cr20	Cr50	D	DCr20	DCr50
H_2_O_2_ decomposing activity (U mg^−1^ protein)	637.9 ± 180.7^a^	472.7 ± 122.9	285.07 ± 45.5^bd^	231.7 ± 65.7^b^	356.5 ± 109.6 ^cd^	308.3 ± 105.3
SOD (U mg^−1^ protein)	71.72 ± 15.7^ac^	57.54 ± 15.6^bc^	50.58 ± 19.03^bc^	84.7 ± 17.2^a^	59.5 ± 15.8^ab^	55.17 ± 13.19^bc^
GSH (μmol mg^−1^ protein)	0.059 ± 0.016^a^	0.045 ± 0.009^a^	0.047 ± 0.029^a^	0.082 ± 0.05^a^	0.059 ± 0.01^a^	0.076 ± 0.023^a^
Protein carbonyls (nmol mg^−1^ protein)	3.89 ± 0.48^a^	4.31 ± 0.88^a^	4.33 ± 1.77^a^	5.57 ± 1.53^a^	5.45 ± 1.67^a^	5.89 ± 1.38^a^
TΑC (mmol DPPH mg^−1^ protein)	0.171 ± 0.044^a^	0.121 ± 0.031^a^	0.143 ± 0.045^a^	0.229 ± 0.036^b^	0.175 ± 0.047^a^	0.183 ± 0.05^a^

Different superscripts in the same row represent statistically significant differences (*p* < 0.05)

#### Superoxide dismutase (SOD) activity

Crocin, at the higher dose, induced a significant increase of SOD activity in the liver. Diabetes did not cause a statistically significant increase of SOD activity in the liver, although the *p* value was low (0.055). On the other hand, the supplementation of crocin to diabetic rats did not alter the enzyme’s activity to a statistically significant degree. Nevertheless, we consider noteworthy that when comparing the DCr50 group with the diabetic group, the *p* value was as low as 0.078 (Table 2). In the kidney, SOD activity did not increase significantly. However, in groups DCr20 and DCr50 the activity significantly decreased compared to the D group (Table 3).

#### Reduced glutathione (GSH) levels and protein carbonyls

The administration of 50 mg kg^−1^ of crocin to healthy animals increased GSH concentration in the liver (*p* < 0.01), while the administration of the lower dose decreased the concentration in the kidney (*p* < 0.05). Nevertheless, no diabetic group was influenced regarding the concentration of protein carbonyls or GSH in any organ (Tables 2 and 3). That said, it is noteworthy that regarding protein carbonyls our results had a relatively high standard deviation, which makes difficult their interpretation.

#### Total antioxidant activity (TAC)

Total antioxidant activity in the liver significantly increased in the Cr50 group (*p* < 0.05). The D group showed a rising trend in hepatic TAC compared to healthy animals, albeit marginally non-significant (*p* = 0.078). The DCr20 group did not present any significant alterations compared with the D group, but the DCr50 group showed a significantly decreased TAC value compared with the diabetic controls (Table 2). In the kidney, diabetes increased TAC significantly and the administration of crocin at the dose of 20 mg kg^−1^ reversed to some degree this effect, but not in a statistically significant manner (*p* = 0.078)

### Effect of crocin intake on PAI-1 activity

**Fig. 2:**
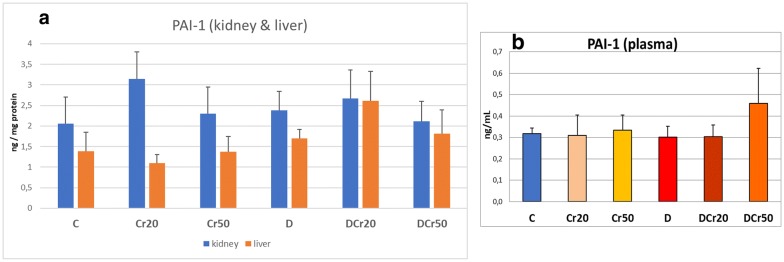
PAI-1 activity in **a** kidney and liver and **b** plasma (mean ± SD)

The activity of PAI-1 in plasma was not affected, neither in the Cr20 and Cr50 groups, nor by the diabetic state or the subsequent crocin administration. In the liver, diabetes did not alter significantly PAI-1 activity. In the kidney, crocin-treated diabetic groups did not show statistically significant changes compared to the D group. The results are illustrated in Fig. 2.

### Effect of crocin on gene expression

**Fig. 3:**
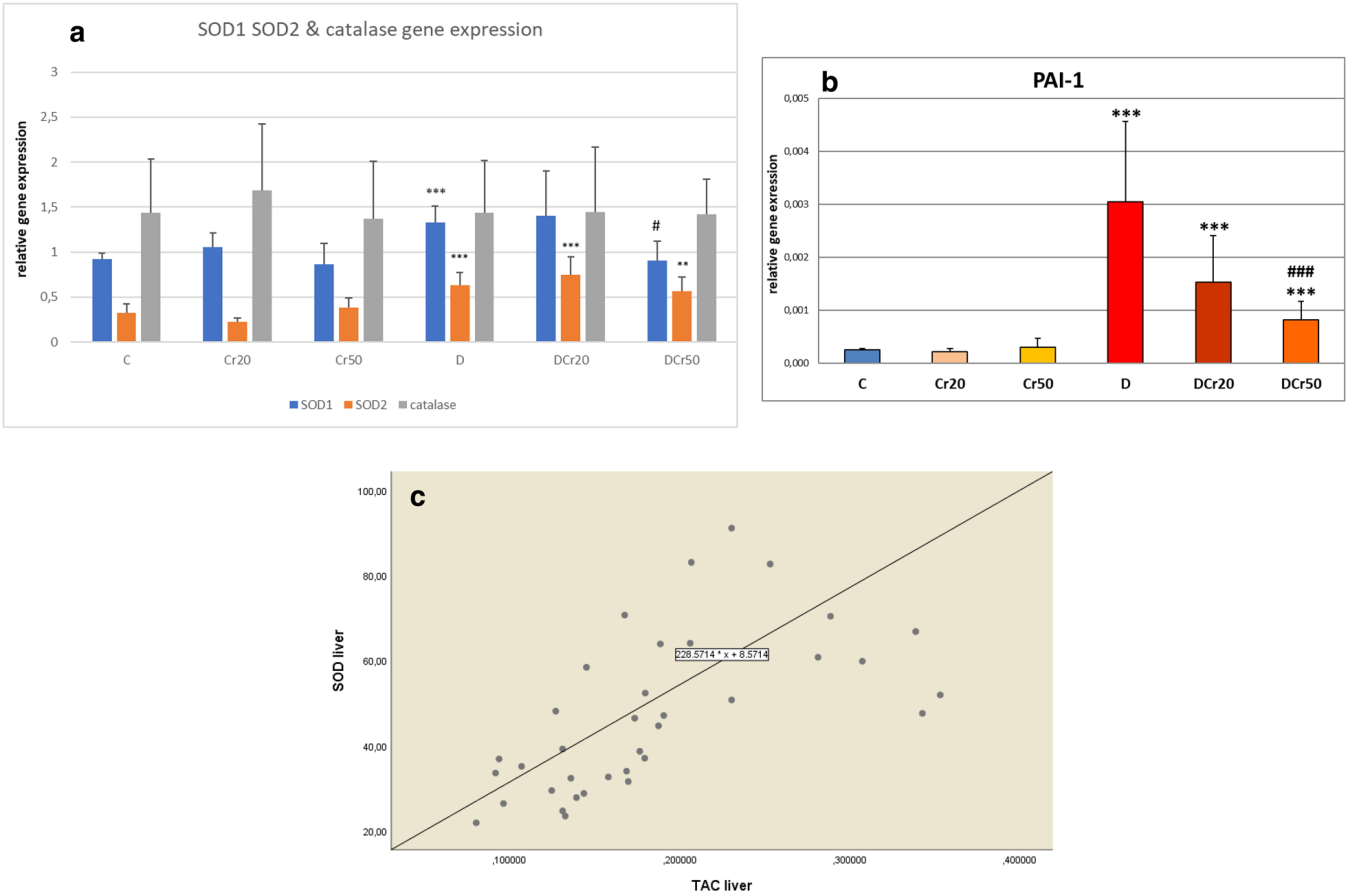
**a** Gene expression of SOD1, SOD2 and catalase in the liver (mean ± SD). Double asterisk (*p* < 0.01) and triple asterisk (*p* < 0.005) denote statistically significant difference compared to the C group, ^#^Statistically significant compared to the D group (*p* < 0.05). **b** Gene expression of PAI-1 gene in the liver (mean ± SD). The triple asterisk (*p* < 0.005) denotes a statistically significant difference compared to the C group; ^###^Statistically significant compared to the D group (*p* < 0.005). **c** The strong correlation between SOD activity and TAC in liver (r_s_ = 0.732, *p* < 0.001)

Αstatistically significant increase of the SOD1 gene expression in the liver was noticed in the D group compared to the controls (*p* < 0.01).The administration of 20 mg kg^−1^ crocin did not affect the expression of SOD1 gene in diabetic animals but, interestingly, this was accomplished with the 50 mg kg^−1^ dose (*p* = 0.01) (Fig. 3a).

Furthermore, diabetes affected the SOD2 gene by increasing significantly its expression in the liver, compared to the control group (*p* < 0.01).However, crocin administration did not alter this effect (Fig. 3a).

Finally, diabetes induction or subsequent treatment with crocin did not affect catalase gene expression in the liver (Fig. 3a).

Regarding the PAI-1 gene expression in the liver, it was significantly increased in the D group compared to the controls (*p* < 0.01). Crocin at the dose of 20 mg kg^−1^ induced a salient trend of amelioration of this effect (*p* = 0.055), while the 50 mg kg^−1^ dose induced a statistically significant alleviation (Fig. 3b).

## Discussion

The present study involved for the first time the effect of crocin supplementation on PAI-1 activity in liver, kidney and plasma, as well as on the expression of SOD1, SOD2, catalase and PAI-1 genes. Although the effect of crocin administration on the antioxidant status of streptozotocin-induced diabetic rats is studied in several publications, only a small part of this literature studies the effect of the per os crocin administration [14–16].

Regarding the effect of crocin administration on glucose levels in the blood of healthy rats, Tamaddonfard et al. [17] reported no hypoglycemic effect. On the contrary, Arasteh et al. [18] reported that the intraperitoneal administration of a hydromethanolic saffron extract does have such an effect. In our study, glucose levels were decreased by crocin in both Cr20 and Cr50 groups, compared to controls. One might point out that there is a discrepancy between our results and the results by Tamaddonfard et al. [17]. Nevertheless, it is important to comment that when crocin is administered per os, as in this study, it is converted to crocetin and absorbed as such [19]. Therefore, our protocol is closer to that of Arasteh et al. [18], since they administered a saffron extract, which obviously contained both crocin and crocetin. As for a possible mechanism explaining the effect on blood glucose levels, given that crocin inhibits pancreatic lipase [20], it is possible that it also reduces lipid absorbance and inhibits gluconeogenesis. Regarding the effect of crocin on glucose levels in diabetic rats, we report no statistically significant outcome. Although Altinoz et al. [8] showed a decrease of glucose levels by 13%, they started treatment just 3 days after the injection of STZ, while our animals received crocin 2 weeks later. Such a time interval allows for a more stable picture of the damage inflicted to the pancreas, as reflected in glucose levels.

The available literature does not allow for a definite conclusion regarding the effect of crocin supplementation on body weight. While Hazman et al. [21] reported a weight decrease of overweight rats, in another study [22], the addition of crocin to a high-fat diet for rats induced a decrease in total as well as epididymal fat, but not in animal weight. In our experiment, no statistical differences were detected, yet *p* values were very low (0.059 for the Cr20 group and 0.1 for the Cr50 group). On the other hand, although the induction of diabetes expectantly caused a significant decrease in animal weight, crocin administration did not compensate for this effect, which is in accordance with Altinoz et al. [14] and Hazman et al. [21]. Most probably, the simple administration of an antioxidant such as crocin cannot compensate for the serious weight loss caused by diabetes, at least when insulin levels remain compromised.

The H_2_O_2_ decomposing activity in the liver did not change by the administration of crocin to healthy animals. This result agrees with Magesh et al. [23] and Rahbani et al. [24]. Furthermore, in our experimentation, the induction of diabetes did not induce any significant change of hydrogen peroxide decomposing activity in the liver. Although the H_2_O_2_ decomposition rate does not exclusively reflect CAT, this enzyme comprises a major element of the H_2_O_2_ decomposing activity. Therefore, the stable H_2_O_2_ decomposition rate agrees with the unaffected expression levels of the catalase gene, also noticed in our experiment. These results are supported by Maritim et al. [25], who suggested that hepatic catalase activity is not consistently altered by a diabetogen (e.g. STZ), nor by the administration of some antioxidants such as melatonin, quercetin or various vitamins.

To our knowledge, our manuscript presents for the first time the effect of long-term crocin administration on the H_2_O_2_ decomposing activity in the kidney. Boussabbeh et al. [26] injected i.p. a single dose of crocin (250 mg kg^−1^) and reported no alterations in catalase activity. In our experiment, the Cr50 group exhibited a significantly lower H_2_O_2_ decomposing activity compared to the controls, while the Cr20 group did not. Carotenoids can be beneficial at low concentrations but harmful at high concentrations (prooxidant action), especially when not administered along with other substances [27]. In the previously mentioned review, Maritim et al. [25] state that renal catalase activity exhibits a clearer decrease in diabetes, an element reported by Kakkar et al. [28] and identified in our experiment as well. On the other hand, the administration of antioxidants may alleviate this effect [5]. Such an alleviation did occur in our experiment, but only with the 20 mg kg^−1^ crocin dose. While it is not always clear why antioxidants do not necessarily work in a dose-dependent manner, it is true that in an oxidative stress state (e.g. diabetes), antioxidant enzymes may be depleted. In this state, antioxidant substances such as crocin can reduce oxidative stress by scavenging ROS and contribute to some extent to the restoration of enzyme levels.

In the only study that relates to our work, Magesh et al. [23] administered i.p. crocetin with no effect on SOD activity in the rat liver. In our experiment, the administration of crocin to healthy animals increased SOD activity, but only in the DCr50 group. According to Wang et al. [29], such an increase is possibly due to an increase in Νrf2 (nuclear factor-erythroid 2-related factor 2) activity, which has an important role in the cellular defense against oxidative stress and a proven relationship with the increase of antioxidant enzyme activity after the administration of antioxidants such as vitamin A. On the other hand, various research teams report contradicting results regarding the effect of diabetes induction on the activity of SOD in the liver [e.g. 25], although they always reverse these results by administering some antioxidant other than crocin. In 2011, Rahbani et al. [24] reported a decrease of SOD activity by the induction of diabetes, a finding that relates to our work since they counterbalanced this decrease by administering a saffron ethanolic extract. Our experimentation only adds extra information to the debate: diabetes tended to increase SOD activity (*p* = 0.055), and this was reversed by the higher crocin dose (once again, only as a trend, *p* = 0.078).

According to Maritim et al. [25], the effect of diabetes on SOD activity in the kidney is not consistent either. In our experiment, the enzyme activity did not change after the induction of diabetes. This result goes along with Maritim et al. [30], as well as with Bandegi et al. [31]. On the other hand, crocin administration decreased renal SOD activity in diabetic animals. Kataya and Hamza [32] presented a similar effect by administering an antioxidant extract of red cabbage.

The concentration of protein carbonyls (liver and kidney) did not change in any group, diabetic or not. In a similar way, in the only previous study on the effect of crocin on healthy animals, El-Beshbishy et al. [33] reported no alteration of the protein carbonyls concentration after the i.p. administration of the substance. Regarding the kidney, Palsamy and Subramanian [34] administered per os resveratrol to healthy rats with no effect on protein carbonyls concentration. Diabetes tends to increase the concentration of protein carbonyls in the rat liver [35, 36] due to the formation of active carbonyl forms. Our findings do not confirm these reports, but this could be just due to the high standard deviation of our results.

The concentration of GSH in the liver of healthy animals was increased by the administration of 50 mg kg^−1^ of crocin. This is in accordance with Gedik et al. [37], and verifies the results reported in 2004 by Ochiai et al. [38], where crocin administration increased GSH concentration in PC-12 cells. Skrzydlewska et al. [39] specifically reported for the rat liver an induction—at gene level—of enzymes that metabolize drugs after the administration of tea polyphenols. Alternatively, such an increase in enzyme activity could be attributed to (a) an increase in the activity of enzymes responsible for GSH synthesis (e.g. g-glutamylcystein ligase or GSH synthetase), or (b) an increase in glutathione reductase activity, which resynthesizes GSH through its oxidized form [40]. Furthermore, in our experiment, GSH did not change in any organ of the diabetic controls. It seems that at their sacrifice time-point, the animals had neither a severe oxidative stress level to the point of GSH significant reduction, nor a stimulus for a compensatory rise of GSH levels.

As far as hepatic TAC in the liver of healthy rats is concerned, Yuan et al. [41] administered a mixed fruit and vegetable juice for 5 weeks and reported an increase in liver TAC. Our result was similar regarding the Cr50 group. Such an outcome can be attributed to the increased activity of both GSH and SOD. Indeed, Bartosz [42] mentioned that in tissue homogenates, hepatic included, TAC is high due to glutathione. Furthermore, a strong correlation was determined between TAC and SOD (Fig. 3c). Finally, regarding the Cr20 and Cr50 groups, a strong correlation was determined between TAC and GSH as well (r_s_ = 0.736, *p* = 0.001). Nevertheless, such a correlation between TAC and GSH was not confirmed in the diabetic groups. This suggests a negative impact of diabetes induction on the oxidative status. According to Hosseini et al. [43], hepatic TAC is not necessarily altered by the induction of diabetes, nor by the treatment of diabetics with saffron. With reference to the diabetic controls and the DCr20 group, our results agree to the aforementioned authors. However, in our protocol, the DCr50 group exhibited a statistically significant decrease of TAC levels in the liver compared to diabetic controls, an effect which should not be considered as prooxidant since protein carbonyls remained stable. A question that may arise is why TAC in the DCr50 group is low, even though H_2_O_2_ decomposing activity, SOD, GSH and protein carbonyls are not different from the controls. A possible explanation is that TAC comprises not only the parameters we determined, but other molecules as well (e.g. vitamin C, E, etc.). As mentioned previously, the increase of some antioxidants may induce the decrease of others. Indeed, when comparing the diabetic group with the DCr50 group, TAC alterations are paralleled by the changes in SOD activity: although superscript letters in Table 2 do not indicate any statistical difference because *p* values were slightly higher than 0.05, both TAC (*p* = 0.076) and SOD (*p* = 0.068) are increased in the D group, and both return back to normal levels (TAC; *p* = 0.018, SOD; *p* = 0.1) in the DCr50 group. Hence, we believe that SOD does contribute to the low TAC levels in the DCr50 group.

In the kidney of healthy animals, TAC was not affected by the administration of crocin. This result agrees with Nasiri et al. [44], who reported that the administration of antioxidant compounds did not affect renal TAC in healthy animals. The induction of diabetes did not have any significant impact either. In a study which is the closest available to ours, Karamouzis et al. [45] found that TAC levels in the plasma of patients with chronic kidney disease remained stable in any stage apart from stage five. In our protocol, TAC in kidney was indeed unaffected by the diabetic state. According to Kusano and Ferrari [46], it is possible that TAC is not influenced as long as vitamins A and E remain unaffected.

The effect of diabetes on catalase gene expression in the liver is an element not thoroughly studied and the few existent works have no consistent results. In humans, catalase gene expression regulates catalase activity at different levels (transcription, post-transcription, post-translation) [47]. Nevertheless, in our experiment, catalase gene expression was not affected by diabetes or by crocin administration. Our result agrees with Ahmed et al. [48], who showed that the diabetic state does not influence catalase gene expression. In another case, the expression of this gene was downregulated by the diabetic state [49], an effect not compensated for by the administration of antioxidants (e.g. ascorbic, lipoic acid). The differences of the protocols concerning diabetes induction might explain the discrepancies of these results.

As far as SOD1 gene expression in the liver is concerned, there is no consistent effect of the induction of diabetes and/or of the administration of antioxidants. For instance, Sadi et al. [50] found that the diabetic state decreased SOD1 gene expression and this effect was not changed by resveratrol injection. This is in accordance with the administration of crocin in our experiment. Furthermore, Sadi et al. [49] reported that lipoic acid and vitamin C did not alter the expression of this gene. Nevertheless, in our experiment crocin did alter SOD1 gene expression and compensated for the significant increase induced by diabetes.

In our study, the expression of SOD2 gene in the liver did not change in the crocin-treated healthy animals. Oliveras-López et al. [51] carried out a study with mice, which received extra virgin olive oil rich in polyphenols, and reported that SOD2 gene expression in pancreatic islets was not affected. Moreover, resveratrol administration in healthy rats does not alter significantly SOD2 gene expression in the liver [50]. On the contrary, this expression increased in the D group compared to the controls, an observation that partly agrees with Sadi and Güray [52] who presented an increase of expression (albeit non-significant). As for the administration of antioxidants, neither resveratrol [50] nor the injection of lipoic acid [52] altered SOD2 gene expression, in accordance with our outcomes.

Compared to type-2 diabetes, the effect of type-1 diabetes on fibrinolysis is relatively under-researched and probably underestimated, possibly due to the clinical significance of thromboembolism in type-2 diabetes. Nevertheless, there is a consensus that the two different types of diabetes have an overlapping pathophysiology [53]. Regarding the mechanisms involved in the alteration of PAI-1 activity by diabetes, a role for hyperglycemia per se (which applies to type-1 diabetes as well) is strongly suggested [54].

The expression of PAI-1 gene in the liver seems to increase in diabetes mellitus, as well as in several protocols of hepatic injury, and is mainly attributed to mechanisms mediated by glucagon and/or reactive oxygen species [55]. A known pathway of activation of genes, among them PAI-1, includes the phosphorylation of Smad proteins [56]. In that study, the expression of PAI-1 gene was induced by TGF-β and α-lipoic acid inhibits this action in hepatic cells by inhibiting TGF-β-mediated molecular mediators such as Smad3. In our experiment crocin, being an antioxidant, may have acted in a similar way.

Neither the induction of diabetes nor crocin supplementation did affect PAI-1 activity in the liver in any group. The same applies with PAI-1 activity in plasma. Although it might be considered as contradictory that the increase of gene expression was not accompanied by a respective increase of PAI-1 activity, this is not necessarily so. Fibrinolysis involves complex mechanisms and numerous activating and inhibiting factors, and fibrinolytic agents can be readily consumed or inhibited upon release. Additionally, the molecule of PAI-1 has a short half-life of approximately 1 h under physiological conditions [57]. Therefore, our findings might be explained by significant differences in tPA and tPA/PAI-1 ratio between diabetic and normal subjects [58]. Besides, PAI-1 inhibits the action of plasminogen activators such as tPA [9]. That said, a more thorough investigation of the effects of type-1 diabetes on tissue fibrinolysis would be of interest. As far as the administration of antioxidants is concerned, Chan et al. [59] did not notice any significant variation of the plasma PAI-1 activity in diabetic rats after the administration of astaxanthin, an outcome in accordance with our results. The fact that crocin impressively corrected the effect of diabetes on PAI-1 gene expression but had no similar effect on hyperglycemia allows us to suggest that, although the elevated glucose levels might have played a role in the expression of the gene, some other factors that affect gene expression must be involved.

With respect to the kidney, PAI-1 activity is reported to increase in the diabetic state, but this rise accompanies the depletion of GSH [60]. In our experiment, PAI-1 activity in kidney did not increase in the D group, either due to the stable GSH levels in the kidney or because of a compensatory increase in tPA. Indeed, Fisher et al. [61] reported that tPA levels increased in the presence of high glucose concentration in renal mesangial cells. However, an increased PAI-1 activity in the kidney is related to diabetic nephropathy [62].

The activity of ALT in blood serum decreased significantly by the administration of crocin to healthy animals in a dose dependent manner. This finding agrees with El-Beshbishy et al. [33], who reported reduced ALT activity after the i.p. administration of crocin at the dose of 200 mg kg^−1^ for 7 days, as well as with Asdaq and Inamdar [63], who found that that the per os crocin treatment to healthy rats at the dose of 19.34 mg kg^−1^ for 5 days decreased ALT activity as well. Regarding AST activity in serum, the higher dose of crocin resulted in a strong trend of decrease (*p* = 0.065), a result that again agrees with El-Beshbishy et al. [33]. Such a decrease could be due to the antioxidant effect of crocin, a hypothesis supported by Djordjevic et al. [64], who administered another carotenoid (astaxanthin) to football players and found decreased AST activity along with a decrease in the production of reactive oxygen species. Furthermore, the activities of ALT and AST in blood serum significantly increased in the diabetic animals compared to controls. In diabetes, the necessary energy exploitation of amino acids through protein catabolism is achieved via transamination with the assistance of aminotransferases, which are elevated in this case. Referring to our experiment, crocin administration managed to decrease ALT and AST activities in the DCr20 group, whereas the 50 mg kg^−1^ dose did not have the same effect. It is common for an antioxidant to affect some parameters in a positive way at a certain dose, yet have a reverse effect when the dose is higher (probably acting in a prooxidant way). Indeed, Altinoz et al. [14] noticed that crocin at the dose of 20 mg kg^−1^ induced a decrease in ALT and AST activities, while Kianbakht and Hajiaghaee [65] did not detect any decrease after the administration of 50 mg kg^−1^ of crocin.

Regarding BUN, there was a significant increase in the diabetic controls compared to the C group, an element in accordance with Kulina and Rayfield [66]. Diabetes mellitus triggers gluconeogenesis, hence muscle tissue amino acids are mobilized and used as energy source. For their uptake and exploitation by the liver, these amino acids need to be catabolized to alanine. Alanine plays a dual role: as a precursor used for gluconeogenesis, as well as a transporter of nitrogen into the liver, where it is used for the formation of urea [67]. In our experiment, although the decrease induced by either dose of crocin was not statistically significant, the margin was close (*p* = 0.107 in the DCr20 group and *p* = 0.128 in the DCr50 group). From a biological point of view, and given that these results refer to different doses of the same substance, the probability that both null assumptions are wrong should be even lower. Indeed, such an extrapolation is supported by the results of Altinoz et al. [15]. The administration of crocin induced a decrease in ALT activity, which allows for the speculation that protein catabolism decreased, resulting to decreased alanine production and BUN levels. Besides, in a study with diabetic mice [68], the administration of more broadly used antioxidant compounds (i.e. vitamin C, E) did decrease BUN levels. These authors attribute this outcome to the antioxidant and anti-inflammatory effects of these substances.

Oxidative stress in diabetes mellitus is due to increased lipid oxidation and production of reactive oxygen and nitrogen species, and induces direct damage to nephrons giving rise to vasoconstriction, platelet aggregation and cellular toxicity [69]. Possibly, this mechanism of renal damage gradually leads to kidney disease resulting in creatinine accumulation in the blood stream. Creatinine is a simple kidney status biomarker with its concentration being elevated in the blood when there is renal disease [70]. In a series of studies [71–73], various authors reported that crocin administration did not influence creatinine in a few species, rat included. We believe that the statistically significant decrease in the Cr20 group is mainly due to the coincidentally low standard deviations, given that the means are very close. Furthermore, creatinine significantly increased in the D group compared to the C group, and neither dose of crocin could compensate for this increase, results that agree with Altinoz et al. [15].

Regarding cholesterol and triglyceride concentration in blood serum, Asdaq and Inamdar reported that the per os supplementation of crocin diluted in a H_2_O vehicle containing carboxymethylcellulose for 5 days reduced cholesterol and triglyceride levels in healthy rats [63]. In our study, no group of healthy rats exhibited such an effect, but the differences of the two protocols regarding the duration of the treatment and the composition of the vehicle constitute difficult any comparison. The elevated cholesterol and triglyceride levels observed in the diabetic controls agree with Young et al. [74]. Reduced insulin secretion occurring in diabetes mellitus increases lipolysis. Insulin inhibits hepatic VLDL production and promotes the catabolism of lipoproteins, which are rich in triglycerides. On the other hand, crocin did not reduce cholesterol or triglyceride levels, a result that disagrees with Altinoz et al. [75]. This discrepancy could be due to the protocol differentiation: we started crocin treatment 2 weeks after STZ injection, while Altinoz et al. [75] supplemented crocin as early as 3 days after STZ. Our 2-week interval between STZ injection and the beginning of crocin treatment must have induced a more severe potentiation of gluconeogenesis, hence higher levels of cholesterol and triglycerides, an effect that crocin could not reverse.

## Μethods

### Animals

Forty-two 8-week old male albino Wistar rats (230 ± 10 g) were provided by the rat colony of the Labs of Physiology, Pharmacology, Biochemistry and Toxicology, School of Veterinary Medicine, Faculty of Health Sciences, Aristotle University of Thessaloniki, Greece. The rats were kept individually in propylene cages at room temperature (24 ± 2 °C) under a 12-h light-dark cycle and consumed conventional rat chow and tap water. The animals were allowed 1 week to adapt to the individual housing. The experimental procedures were carried out according to EU Directive 2010/63/EU and the local veterinary and scientific authority (Region of Central Macedonia, Directorate General of Rural Economy and Veterinary Medicine, Directorate of Veterinary Medicine, Department of Animal Health, Welfare, Veterinary Drugs and Applications) approved the protocol (371421/3559).

### Induction of diabetes

The animals were randomly chosen as follows: six animals served as controls, 12 animals formed the two groups of negative controls (healthy rats that received either 20 mg kg^−1^ or 50 mg kg^−1^ of crocin) while the rest 24 animals were subjected to type-1 diabetes induction. For this purpose, we administered i.p. 110 mg kg^−1^ b.w. nicotinamide (NA) dissolved in natural saline, and 15 min later 65 mg kg^−1^ b.w. streptozotocin (STZ) dissolved in 10 mM sodium citrate (pH 4) was injected [76], once again i.p. [77]. Streptozotocin is one of the most prominent diabetogens utilised for the study of diabetes and induces diabetes by causing necrosis of the pancreatic β cells [78]. The animals were in a non-fasting state before the administration of the drugs. According to Masiello et al. [76], the nicotinamide injection that precedes that of STZ protects the insulin producing β cells by preventing their complete destruction by streptozotocin, resulting to more stable hyperglycemia. The successful induction of diabetes was confirmed 14 days after STZ administration. Given that, according to our experience, glucose levels tend to fluctuate for a few days after the nicotinamide/STZ injection, the 14-day interval allows for a safer characterization of an animal as diabetic. Glucose was determined using a one-touch glucose meter (Contour Next, Bayer, Germany), following a 4 h-fasting. Animals with a glucose value above 200 mg dl^−1^ were considered as diabetic. Longer fasting periods were avoided, as acute energy limitations increase PAI-1 levels in plasma [79]. The overall diabetes induction success rate was 75%, hence 18 rats composed the three diabetic groups of the study, as described in the following paragraph.

### Experimental protocol

After the induction of diabetes, the animals formed six groups (i.e. three groups of healthy and three groups of diabetic rats). The control group, named as group C (Controls, n = 6), received natural saline and, 15 min later, 10 mM sodium citrate (pH 4) (the two respective vehicles of the injected substances). Crocin, when supplemented, was administered orally. The healthy, crocin-treated groups were named as Cr20 (healthy animals which received 20 mg kg^−1^ b.w. crocin, n = 6) and Cr50 (healthy animals which received 50 mg kg^−1^ b.w. crocin, n = 6). The diabetic groups were named as group D (Diabetic controls, n = 6), DCr20 (Diabetic animals which received 20 mg kg^−1^ b.w. crocin, n = 6) and DCr50 (Diabetic animals which received 50 mg kg^−1^ b.w. crocin, n = 6). Crocin (98% purity, Sigma, St. Louis, Missouri, U.S.A.) was administered daily for 4 weeks. Crocin dosage selection was based on previous work of other research groups. Specifically, Abou-Hany et al. [16] used the dosage of 20 mg kg^−1^ b.w. and found that crocin suppressed diabetic nephropathy, whereas Kianbakht and Hajiaghaee [65] found that the dosage of 50 mg kg^−1^ b.w. has a hypoglycaemic effect. Crocin was administered after being dissolved in drinking water. Specifically, the substance was diluted in 5 ml of drinking water and administered via bottles covered by aluminum foil for photoprotection. Under the above-mentioned conditions (light, pH, air, temperature) the molecule of crocin maintains its stability [80]. The administration of crocin as an aqueous solution via water bottles is a novel method, applied only once [13]. We consider as advantageous the simplicity of the method and the avoidance of potential administration of anesthetics, hence keeping the animal welfare at a high level. The daily administration of any treatment via oral gavage, even when carried out by well-trained staff, constitutes a stress factor for the animals, and it is well established that stress can affect glucose levels. Although stress is alleviated by sedating the animals prior to gavage usage, one should take into consideration that most anesthetics do affect the oxidative status [81]. The animals surely ingested the complete and exact dosage, since we provided the solution individually, and it was consumed in a short period of time (15 min on average). The day diabetes was confirmed (14 days after the STZ injection) was considered as day 1 of the experiment. By the completion of the experimental period, the animals were euthanized by decapitation.

### Blood and tissue collection

Immediately after euthanasia, blood was collected into both EDTA-coated tubes (for plasma) and serum separating tubes (for serum) and immediately centrifuged (1370 g, 10 min, 4 °C). Plasma and serum were frozen at − 80 °C until further analysis. The liver and the left kidney were excised, and the right lobe of the liver was immersed in RNA-later solution (Takara Bio Inc, Shiga, Japan) and stored at − 80 °C for gene expression analysis. The rest of the liver and the entire kidney, after being rinsed in cold normal saline, were immersed in liquid nitrogen and immediately stored at − 80 °C.

Plasminogen activator inhibitor-1 (PAI-1) activity was determined in the plasma, the middle lobe of the liver and the left kidney. Alanine aminotransferase and AST activities, glucose, BUN, creatinine, cholesterol and triglycerides were determined in serum. Redox biomarkers (i.e. hydrogen peroxide decomposing activity, GSH, SOD, protein carbonyls and total antioxidant capacity) were determined in homogenates of the middle lobe of the liver and of the left kidney.

### Blood glucose and biochemical tests

For the confirmation of diabetes induction after streptozotocin administration, blood glucose was determined weekly for a 2-week period. For this purpose, a blood drop was obtained from the tail vein. After sacrifice, blood glucose, as well as ALT, AST, BUN, creatinine, cholesterol and triglycerides were determined spectrophotometrically with an automatic biochemical analyzer (Abbott Laboratories, Architect c8000, Abbott Park, Chicago, Illinois, USA).

### Tissue homogenization

For the determination of the redox biomarkers and of PAI-1 activity, hepatic and renal tissue samples were homogenized as follows (modified from [82]): a small piece of tissue was placed in a mortar, liquid nitrogen was added, and the tissue was cracked and pulverized with a pestle. Subsequently, one part (g) of the ground tissue was homogenized with three parts (ml) of 0.01 M phosphate buffered saline (PBS) (154 mM NaCl, 1.06 mM KΗ_2_ΡΟ_4_ and 2.97 mM Na_2_ΗΡΟ_4_, pH 7.4) and a mix of protein inhibitors was added (1 μΜ aprotinin, 1 mg ml^−1^ leuceptin and 1 mM phenylmethanesulfonyl fluoride, Complete Mini Protease Inhibitor Cocktail Tablets-Roche Diagnostics GmbH, Mannheim, Germany). No protein inhibitors were added in the sample homogenates for the determination of PAI-1 activity. The homogenates were forcefully vortexed, centrifuged (15,000*g*, 5 min, 4 °C) and the supernatants were collected.

### Redox biomarkers

Catalase, glutathione peroxidase and peroxiredoxins synergistically convert hydrogen peroxide to water [83]. These enzymes are antioxidant molecules, and the afore-mentioned process they serve, is defined as H_2_O_2_ decomposing activity.

The determination of hydrogen peroxide (H_2_O_2_) decomposition rate was based on a modification of the method of Aebi [84]. In brief, as for the hepatic samples, 5 μl of homogenate was diluted 112 times in a 0.01 M PBS solution, whereas for the nephric ones, the homogenate was diluted 81 times in the same solution. 40 μl of the diluted homogenates was transferred to plastic test tubes containing 2955 μl of 67 mM phosphate buffer (pH 7.4). The samples were vortexed and subsequently incubated at 37 °C for 10 min. The content was transferred to a quartz cuvette and 5 μl of 30% H_2_O_2_ was added. The absorbance change was monitored at 240 nm for 1.5 min. Hydrogen peroxide decomposing activity was calculated using the Molar Extinction Coefficient (MEC) of H_2_O_2_ (MEC refers to its absorbance at the given wavelength. Its SI unit is M^−1^ cm^−1^. The MEC of H_2_O_2_ is equal to 40 l mol^−1^ cm^−1^).

The assessment of SOD activity was carried out as described by Veskoukis et al. [85], a method based on Aureliano et al. [86]. In brief, 740 μl of 50 mM phosphate buffer (pH 7.8), 100 μl of 0.5 mM xanthine solution, 100 μl of 0.1 mM cytochrome c solution and 10 μl of the homogenate were added to a plastic test tube. The mixture was vortexed, transferred to a cuvette and 50 μl of bovine xanthine oxidase (diluted 1/50 from a 25-Unit stock) were added before the cuvette was rapidly inverted three times and the absorbance was monitored at 550 nm for 1 min. The activity of SOD was determined with the use of the Molar Extinction Coefficient of cytochrome c (its SI unit is M^−1^ cm^−1^. The MEC of cytochrome c is equal to 19.2 l mol^−1^ cm^−1^).

Total antioxidant activity in liver and kidney was determined with the method of Janaszewska and Bartosz (modified from [87]). The homogenates were diluted five more times. Subsequently, 40 μl of the diluted homogenate was added to tubes containing 460 μl of 10 mM phosphate buffer (PB, pH 7.4) and 500 μl of 0.1 M 2,2-diphenyl-1-picrylhydrazyl (DPPH) free radical diluted in methanol. The blank contained 500 μl of PB and 500 μl of the DPPH radical. The samples were vortexed and incubated in a dark room for 60 min at room temperature, then centrifuged (20,000*g*, 3 min, 25 °C), and the absorbance was read at 520 nm.

For protein carbonyls determination, a modified version of the method of Patsoukis et al. [88] was followed. Specifically, 50 μl of 20% TCA was added to 50 μl of either hepatic or renal homogenate. The mix was vortexed and incubated in ice bath for 15-min, then centrifuged at 15,000*g* for 5 min at 4 °C. Subsequently, the supernatant was withdrawn and 500 μl of 14 mM 2,4-dinitrophenylhydrazine (DNPH) dissolved in HCl 2.5 N (for the test tubes) or 500 μl of HCl 2.5 N (for the blank) were added. The samples were then incubated in the dark for 1 h at room temperature. During the incubation and every 15 min, the samples were vortexed. Following the incubation, the samples were centrifuged (15,000*g*, 5 min, 4 °C), the supernatant was removed and 1 ml of 10% TCA was added with subsequent vortexing and centrifugation (15,000*g*, 5 min, 4 °C). The supernatant was removed and a mix of 1 ml 1:1 v/v ethanol and ethyl acetate was added before the samples were vortexed and centrifuged (15,000*g*, 5 min, 4 °C) again. The last step was repeated twice. The resulting supernatant was removed, 1 ml of 5 M urea (pH 2.3) was added and the samples were vortexed and incubated at 37 °C for 15 min. Finally, a last centrifugation was carried out at 15,000*g* for 3 min at 4 °C and the absorbance was read at 375 nm. For the protein carbonyls calculation, we used the molar extinction coefficient of DNPH (its SI unit is M^−1^ cm^−1^. The MEC of DNPH is equal to 22 l mol^−1^ cm^−1^).

Reduced glutathione was determined with the method of Reddy et al. [89], as modified by Papadopoulou et al. [40]. Briefly, 20 μl of tissue homogenate, treated with equal volume of 5% TCA, was mixed with 660 μl of 67 mM sodium potassium phosphate (pH 8.0) and 330 μl of 1 mM 5,5-dithiobis-2-nitrobenzoate (DTNB). After incubation of the aliquots for 45 min in the dark at room temperature, the absorbance was read at 412 nm. The calculation of GSH concentration was based on a calibration curve made with the use of commercial standards.

### Determination of total protein concentration

Total protein concentration of the homogenates was determined according to Bradford [ [90], modified]. Specifically, 20 μl of tissue homogenate was added to 1000 μl of Bradford reagent. The blank contained 20 μl of PBS instead of tissue homogenate. The mixture was incubated at 37 °C for 15 min and the absorbance was read at 595 nm. Protein concentration was determined based on a standard curve made of albumin solutions with known concentrations.

### PAI-1 activity

For the determination of PAI-1 activity, the liver and kidney tissue homogenates were further diluted to a final concentration of 1 and 0.2 mg ml^−1^, respectively. The activity in tissue homogenates and in plasma was determined using a commercial ELISA kit [Rat plasminogen activator inhibitor 1 (PAI-1) ELISA Kit (Cusabio, Maryland, USA)]. The assay was carried out following the instructions of the manufacturer. The absorbance was measured with an ELISA plate Humanreader (HUMAN Diagnostic Systems, Germany).

### Quantitative gene expression analysis

The extraction of total RNA was carried out using a NucleoSpin Total RNA Isolation kit (Macherey-Nagel, Duren, Germany), following the instructions of the manufacturer. The RNA concentration was determined spectrophotometrically at 260 nm (Eppendorf BioPhotometer, Eppendorf, Hamburg, Germany) and the samples were stored at − 80 °C until further analysis. For the synthesis of complementary DNA we used the PrimeScript 1st Strand cDNA Synthesis kit (Takara, Bio Inc., Japan) following the instructions of the manufacturer. Quantitative expression analysis was carried out with real-time polymerase chain reaction (RT-PCR), based on the SYBR Green chemistry. The Glyceraldehyde 3-phosphate dehydrogenase (GAPDH) housekeeping gene served as internal control. Primer3 input software (version 0.4.0) [91] was used for primer design based on nucleotide sequences available in GenBank (Accession numbers-ΡΑΙ-1: M24067.1, SOD1: BC082800.1, SOD2: NM_017051.2 Catalase: NM_012520.2). Table 4 presents the sequences of the primers and the amplicon sizes.

**Table 4:** Primers used for gene expression analysis

Primer	Sequence (5ʹ–3ʹ)	Amplicon size (bp)
ΡΑΙ-1-F ΡΑΙ-1-R	ATCAACGACTGGGTGGAGAG GAAATAGAGGGCGTTCACCA	108
SOD1-F SOD1-R	GGTGAACCAGTTGTGGTGTCAGG ATGAGGTCCTGCAGTGGTACAG	114
SOD2-F SOD2-R	TAACGCGCAGATCATGCAGCTG AGGCTGAAGAGCAACCTGAGTT	133
CAT-F CAT-R	CTTCATCAGGGATGCCATGT CGGTCGCTGAACAAGAAAGT	150
GAPDH-F GAPDH-R	CATCACTGCCACTCAGAAGACTG ATGCCAGTGAGCTTCCCGTTCAG	153

For the amplification of PCR, the 20 μl reaction mixture included 2 μl of cDNA, 1× KAPA SYBR FAST qPCR mix (KAPA BIOSYSTEMS, Wobum, MA, USA) and 250–350 nM of each primer pair. A Bio-Rad MiniOpticon System (Bio-Rad Laborarories, Hercules, CA, USA) was used for temperature cycling, which included 40 cycles of the following stages: (a) denaturation for 10 s at 95 °C, (b) annealing/extension for 20 s at temperatures varying from 57 to 63 °C. Every PCR reaction started with a denaturation temperature of 95 °C for 3 min and ended with successive readings between 65 and 95 °C (increment of 0.5 °C) for the generation of a melting curve and the verification of the product specificity. The gene expression quantification was carried out with the comparative Ct method, also known as the 2^−ΔΔCt^ [92].

### Statistical analysis

Data were processed with the Kruskal–Wallis test and pairwise comparisons were performed with the Mann–Whitney U test. Statistical correlations were established with the Spearman’s rho. Statistical significance level was set at *p* < 0.05. All experimental data were analyzed with the SPSS version 24.0 (IBM Corp., Armonk, New York, USA) and are presented as mean ± SD.

## Conclusions

In conclusion, crocin treatment in NA-STZ-induced diabetic rats significantly affected hepatic transaminases ALT and AST, SOD1 and PAI-1 gene expression, as well as nephric H_2_O_2_ decomposing activity. Additionally, crocin was associated with ameliorating trends regarding BUN, hepatic SOD activity and TAC levels in the liver. The exact mechanism of action of crocin, with respect to the parameters studied in our protocol, is not always well established. Although, the investigation of each particular mechanism should be the subject of further research, we believe that our results—especially regarding gene expression—contribute to this promising field.

## Supplementary information

**Additional file 1.** Altogether data for JBR.

## Data Availability

All data generated or analysed during this study are included in this published article and its Additional file 1.

## Abbreviations

ALT: Alanine aminotransferase
AST: Aspartate aminotransferase
BUN: Blood urea nitrogen
C: Control group
Cr20: Healthy animals which receives 20 mg kg^−1^ bw crocin
Cr50: Healthy animals which received 50 mg kg^−1^ bw crocin
D: Diabetic control group
DCr20: Diabetic animals which received 20 mg kg^−1^ bw crocin
DCr50: Diabetic animals which received 50 mg kg^−1^ bw crocin
DNPH: 2,4-Dinitrophenylhydrazine
DPPH: 2,2-Diphenyl-1-picrylhydrazyl
DTNB: 5,5-Dithiobis-2 nitrobenzoate
EDTA: Ethylenediaminetetraacetic acid
GAPDH: Glyceraldehyde 3-phosphate dehydrogenase
GSH: Reduced glutathione
LPS: Lipopolysaccharide
NA: Nicotinamide
PAI-1: Plasminogen activator inhibitor-1
PBS: Phosphate Buffered Saline
PCR: Polymerase chain reaction
SOD: Superoxide dismutase
SOD1: Cytoplasmic copper/zinc superoxide dismutase
SOD2: Mitochondrial manganese superoxide dismutase
STZ: Streptozotocin
TAC: Total antioxidant capacity
TCA: Trichloroacetic acid
tPA: Tissue plasminogen activator
